# Autoimmunity and tumor immunology: two facets of a probabilistic immune system

**DOI:** 10.1186/s12918-014-0120-4

**Published:** 2014-11-11

**Authors:** Jaime Iranzo, Pablo Villoslada

**Affiliations:** Centro de Astrobiología, INTA – CSIC, Madrid, Spain; Institute of Biomedical Research August Pi Sunyer (IDIBAPS), Hospital Clinic of Barcelona, Casanova 145, Cellex Center 3A, 08036 Barcelona, Spain; Current address: National Center for Biotechnology Information, National Library of Medicine, National Institutes of Health, Bethesda, MD USA

**Keywords:** Comparative immunology/evolution, Autoimmunity, Tumor immunity, Antigen presentation/processing, Mathematical modeling

## Abstract

**Background:**

The immune system of vertebrates has evolved the ability to mount highly elaborate responses to a broad range of pathogen-driven threats. Accordingly, it is quite a challenge to understand how a primitive adaptive immune system that probably lacked much of its present complexity could provide its bearers with significant evolutionary advantage, and therefore, continue to be selected for.

**Results:**

We have developed a very simple model of the immune system that captures the probabilistic communication between its innate and adaptive components. Probabilistic communication arises specifically from the fact that antigen presenting cells collect and present a range of antigens from which the adaptive immune system must (probabilistically) identify its target. Our results show that although some degree of self-reactivity in the immune repertoire is unavoidable, the system is generally able to correctly target pathogens rather than self-antigens. Particular circumstances that impair correct targeting and that may lead to infection-induced autoimmunity can be predicted within this framework. Notably, the probabilistic immune system exhibits the remarkable ability to detect sudden increases in the abundance of rare self-antigens, which represents a first step towards developing anti-tumoral responses.

**Conclusion:**

A simple probabilistic model of the communication between the innate and adaptive immune system provides a robust immune response, including targeting tumors, but at the price of being at risk of developing autoimmunity.

## Background

For more than a century, the immune system (IS) of vertebrates has been admired by biologists for its versatility and stunning complexity, which continues to impress as new findings are made in the field. The plethora of cell types, molecular interactions and soluble factors that are involved in the immune response is not only responsible for an organisms defense against infections but also, they play an important role in self-maintenance and antitumor responses [1]. The complexity that is currently observed in the IS of vertebrates is the consequence of an evolutionary process that started more than 500 million years ago [2–4]. Therefore, primitive stages of the IS probably lacked many of the current cell types, lymphoid organs and regulatory mechanisms [5], although they still had to cope with the risk of potential autoimmunity. Understanding how this simpler IS was sufficiently functional to persist and continue evolving under the pressure of natural selection constitutes a challenge for immunologists and evolutionary biologists alike [6]. In a discipline mainly driven by experimental work, simple theoretical models can be used to provide an intuitive understanding of how the IS works, as well as the conceptual frameworks necessary to organize and integrate experimental findings into a coherent system [7]. Moreover, as new forms of IS are being discovered in organisms other than vertebrates, theoretical studies on the very general properties of the vertebrate IS may help us to formulate hypotheses on what we might expect for other such IS’s [8].

One of the first conceptual models of the IS was proposed more than fifty years ago and based the immune response firmly on the foundations of self vs. non-self discrimination [9]. According to a two-signal model developed subsequently [10], discrimination between self and non-self could be achieved if two concurrent triggers were required in order to induce a response. While these models emphasized the importance of correct Antigen (Ag) targeting, they could neither explain tolerance towards certain non-self Ags, nor the responses against self Ags. The danger model introduced in the nineties [11], integrated those phenomena by establishing that the IS must be activated by danger signals in order to initiate a response. Conversely, Ags (self or non-self) that are presented in a non-danger context become tolerated. While focusing on the context required for IS activation, the danger model does not directly address the question of specific Ag targeting (i.e. how the IS decides, in a context of danger, which particular Ag should be targeted). A different perspective was adopted in the tunable activation threshold model [12] in which it was argued that the IS actively tracks Ag abundance and responds against Ags whose abundance changes abruptly [13,14]. Situating the detection of sudden changes in Ag as a central element in the immune response has recently been reformulated as the discontinuity theory of immunity [7]. From a more abstract point of view, the ability of the IS to detect and manage changes in the self was the basis of the cognitive IS theory adopted in the late nineties [15].

Since the discovery of autoimmune diseases and anti-tumor immunity [16,17], self-reactivity has become a central issue that all theoretical models of the IS must deal with. One particularly interesting question is whether such phenomena are exceptional (e.g. due to the malfunction of the IS) or if they are simply natural outcomes displayed by any conceivable IS [18]. We have developed a simple, conceptual model that aims to integrate the basic features of the IS into a unified framework. Inspired by evolutionary considerations, we hypothesize that a simplified model of the IS, even if it neglects some degree of its actual complexity, can shed some light on the intrinsic properties of the immune response. Likewise, such a model might provide hints as to the minimal elements required for an IS to be functional.

**Figure 1:**
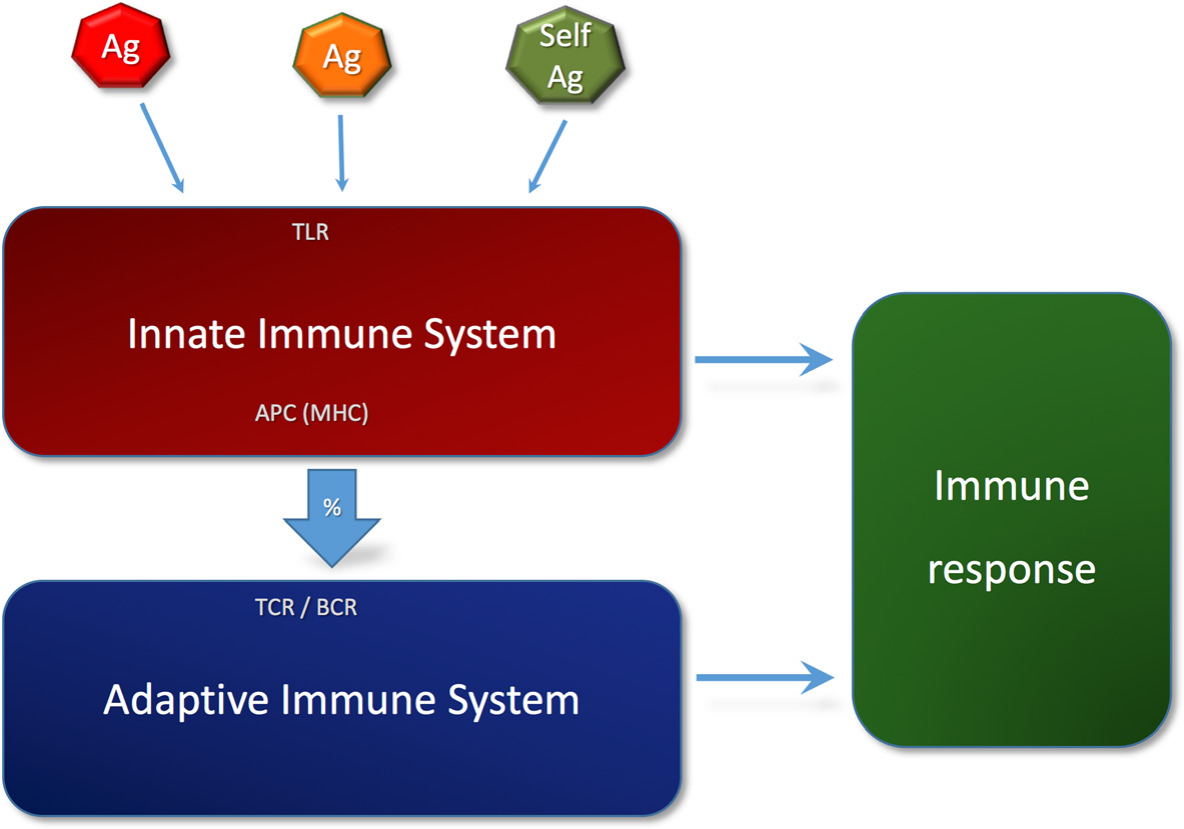
**Conceptual scheme of the immune response.** Ags are collected from the environment by specific cells (APCs) that belong to the IIS. Following activation of the IIS by danger signals, probabilistic communication between the IIS and AIS occurs via Ag presentation, permitting the AIS to mount an Ag-specific immune response. The overall immune response is the result of innate and adaptive effector mechanisms.

In order to construct our model, we focused on the communication between the innate and adaptive components of the IS. On the one hand, the innate immune system (IIS) has evolved to recognize conserved molecular patterns [19] that are usually associated to infections or tissue damage – the so-called danger signals. Once such signals are detected, the IIS is activated and it sets in motion an innate response [20]. Conversely, the adaptive immune system (AIS) can recognize virtually everything, although it must be activated by Ag-presenting cells (APCs) that pertain to the IIS. Communication between the IIS and AIS, materialized in the process of Ag presentation, aims to ensure that the adaptive immune response is targeted to the correct Ag (Figure 1). Accordingly, the key property of IIS-AIS communication must be highlighted. In a context of danger, the target Ag is presented by APCs together with a set of non-target Ags from the environment. Because danger signals need not be physically linked to the target Ag (e.g., they may be endogenous factors produced by damaged tissue) there is no way to identify such Ag in a deterministic way. In other words, the IS must choose its target based on incomplete context information, a decision-making process under uncertainty. Control mechanisms, such as central and peripheral tolerance, are necessary to enhance the probability of targeting the correct Ag and it is this probabilistic nature of IIS-AIS communication that we aim to capture with our model.

Classical explanations of how infectious agents may provoke autoimmunity [21] fit into the aforementioned conceptual framework at three levels: first, through the incorrect activation of the IIS – bystander activation; second, through a failure in IIS-AIS communication – epitope spreading; and third, through a non-specific adaptive response – molecular mimicry and superantigens. To account for the first two situations, our model allows the role that central and peripheral tolerance play in minimizing the risk of autoimmunity to be evaluated qualitatively. In addition, it shows that some classes of autoimmunity and antitumor responses are related outcomes that arise from the probabilistic nature of IIS-AIS communication.

The rest of the article is structured as follows: we begin Results section by describing the main ingredients of the model, which include an antigenic microenvironment, central tolerance through negative selection, and a simple form of peripheral tolerance. Then we present the results of the mathematical analysis of the model in what concerns self-reactivity, pathogen targeting and tumor detection. When possible, order of magnitude estimations are given to illustrate the performance of the IS in different scenarios. The Discussion highlights the relevance of our results in the context of autoimmunity and tumor immunology, and explores the possibility that the probabilistic model can be extended to non-vertebrate IS’s. A comprehensive exposition of the model and its analysis is provided in Methods.

## Results

### Probabilistic model of the IS

We set out to model IIS-AIS communication as a random process based on the following principles: (i) Antigens, foreign and self, are collected randomly by APCs in a non-selective manner; (ii) APCs sense pathogens or their effects on the environment (e.g. responses provoked by tissue damage) and they are activated; (iii) Ag presentation by active APCs is probabilistic, such that the probability of presenting an Ag depends on its abundance and the affinity of the MHC: Major Histocompatibility Complex-peptide complex expressed on the surface of the APC. For the sake of simplicity, we refer to the conjunction of both these factors (abundance and affinity) through the term “effective abundance”. While inspired by the immune system of vertebrates, our intention was to keep the model sufficiently general so that it could be applied to other hypothetical immune systems with an adaptive component. Therefore, we did not consider further details regarding the specific cell types and molecular interactions involved in the immune response, which would decrease the opportunity to perform specific predictions.

The first step towards building a probabilistic model of the IS lies in defining the antigenic microenvironment in which the IS exists. In a healthy scenario, the antigenic microenvironment can be envisioned as a set of self-Ags, *A = {A*_*1,*_*…, A*_*i,*_*…, A*_*N*_*}*, each with a particular effective abundance *a*_*i*_. In analogy to the molar fractions in Chemistry, effective abundances were normalized so that they added up to one. Thus, *a*_*i*_ is the probability of randomly choosing the Ag *A*_*i*_ in the full set *A*. Pathogens alter the antigenic microenvironment by introducing additional pathogen-associated Ags, *P = {P*_*N+1,*_*…, P*_*M*_*}*, whose effective abundances equal *p*. Since the sum of all effective abundances must be equal to one, the presence of a pathogen modifies the effective abundance of the self Ags, which become *a’*_*i*_ *= (1 - p) a*_*i*_. If desired, non-pathogenic, non-self Ags (for instance, those belonging to commensal gut microbiota) could be included in the model as a third set of Ags with their corresponding abundances, although we did not analyze their instance in this particular model.

The next element in the model was central tolerance. We modeled induction of central tolerance as a stochastic process that mimics thymic negative selection. For each Ag in the set of self-Ags we calculated the probability that a naive, self-reactive cell does not see its cognate Ag during a number *t*_*0*_ of presentations that are associated to the maturation process (see Methods). This resulted in an expression that links the effective abundance of a self-Ag, *a*_*i*_, to the probability of it becoming tolerated, *f*_*i*_, whereby:

1 $$ {f}_i=1-{e}^{-{a}_i{t}_0} $$

**Figure 2:**
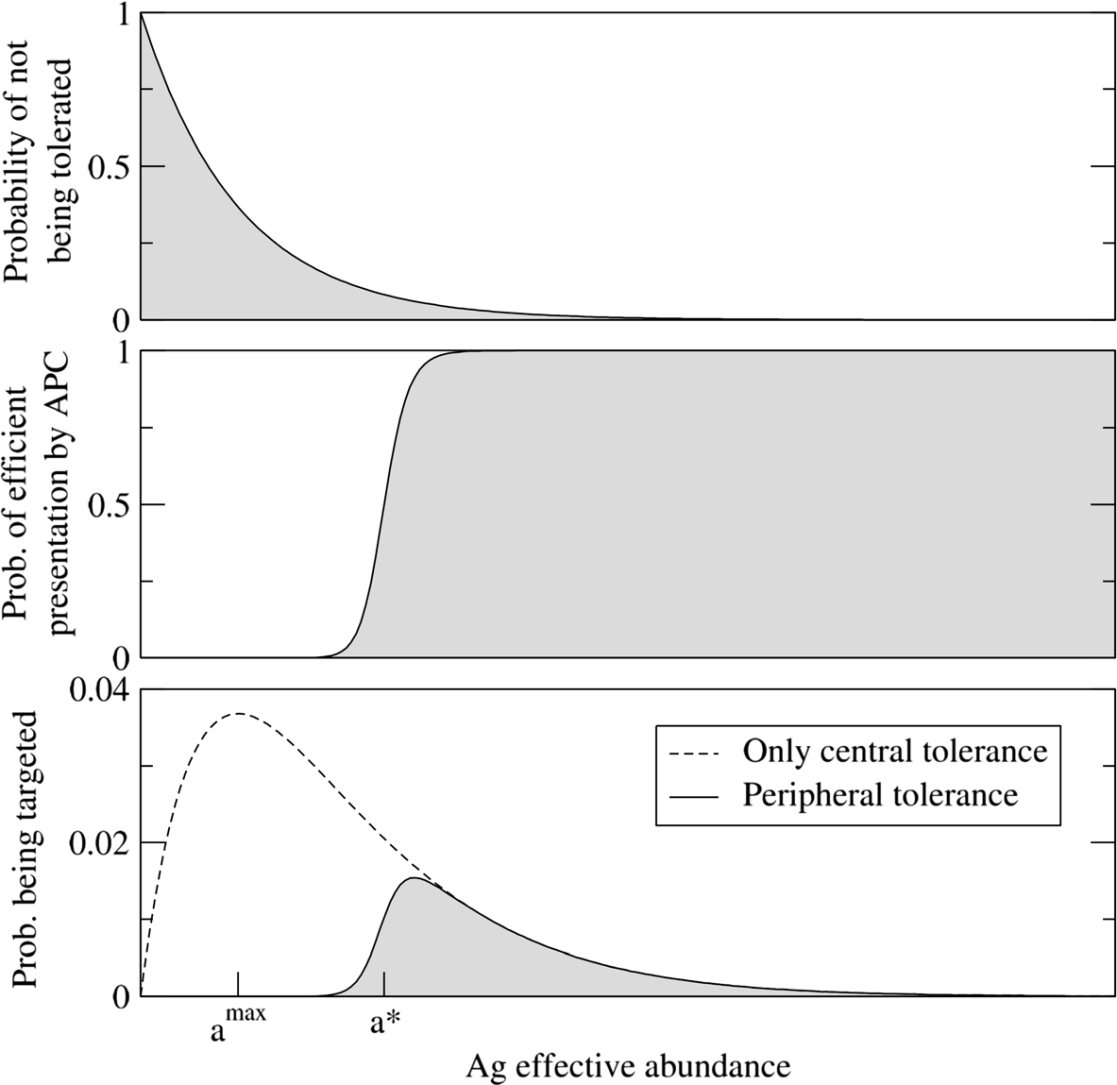
**Effect of central and peripheral tolerance on immune self-reactivity.** The x-axis represents the effective abundance of a self-Ag, in arbitrary units. Top: probability that self-reactive lymphocytes towards a given self-Ag remain after the induction of central tolerance. The curve corresponds to *1 – f*_*i*_ from eqn. 1 and it can be also interpreted as the fraction of self-Ags with a given effective abundance that remain untolerated. Middle: probability that an Ag (self or non-self) is efficiently presented by an APC. Bottom: combined effect of central and peripheral tolerance on the probability of targeting a self-Ag. In our simple model, peripheral tolerance is determined by the threshold *a* ^*^, the minimum effective abundance required for efficient Ag presentation. In the absence of peripheral tolerance, the probability of targeting a self-Ag becomes greatest for those self-antigens with effective abundances close to *a* ^*max*^.

In this equation, abundant Ags become tolerated but self-reactivity against rare Ag persists (Figure 2, top). In accordance with intuitive expectations, tolerance increases with *t*_*0*_, the number of presentations involved in negative selection.

In recent years several new mechanisms associated with the maintenance of peripheral tolerance have been described, involving regulatory T and B cells, tolerogenic dendritic cell presentation, inhibitory and pro-apoptotic co-stimulatory signals, etc. [22]. However, as our aim was to build a model that was as simple as possible, and from an evolutionary perspective, we hypothesized that a much simpler implementation of peripheral tolerance might be sufficient to yield a functional IS. Several mechanisms have been described to carry out this function: on the one hand, minimal stimulation through the TCR: T cell receptor-Ag interaction must be received to activate the lymphocyte [23–25]; on the other, anergic and regulatory T cells [26,27] exert an inhibitory role, preventing the triggering of responses against low concentration Ags. These findings suggest that a new source of regulation should be introduced into the model, namely the need for a minimum number n* of MHC-peptide complexes at the surface of the APC in order to induce lymphocyte activation. Since the frequency at which a given Ag is presented depends on its effective abundance, this minimum number of MHC-peptide complexes can be translated into an effective abundance threshold a* (see Methods). Accordingly, only those Ags with effective abundances greater than a* are presented efficiently and induce an adaptive response, whereas Ags with sub-threshold abundance can still be presented but they do not produce a response (Figure 2, middle). This approach to modeling efficient Ag presentation constitutes a simple yet functional form of (unspecific) peripheral tolerance, because it controls lymphocyte activation avoiding autoimmune responses against rare, non-centrally-tolerated Ags. It should be kept in mind, though, that peripheral tolerance in vertebrates is an active and complex process that fine-tunes the immune response through multiple regulatory interactions.

### Remaining self-reactivity in the immune repertoire

The probabilistic model allows the effect that tolerance mechanisms exert on the immune repertoire to be evaluated. While negative selection removes lymphocytes that are highly reactive towards a set of self-Ags, rare self-Ags may not be centrally tolerated if they are not confronted by lymphocytes that are reactive to them. In conditions where the IIS is active (e.g. as a result of an ongoing infection or tissue damage), those self-Ags with an effective abundance above the efficient presentation threshold are susceptible to induce immune responses. The probability that this occurs depends on the abundance of each self-Ag and specifically, it is the product of the effective Ag abundance and the probability of not being tolerated. Like very rare self-Ags, common ones are considered to be at low risk of inducing immune responses, essentially because the former are hardly ever presented and the latter are generally tolerated. Rather, the model predicts a maximum reaction risk for self-Ags whose effective abundances lie around a particular value *a*^*max*^ (Figure 2, bottom). Moreover, if central tolerance is complemented with peripheral tolerance (as described in Methods) the risk can be greatly minimized. The only condition is that the abundance threshold for efficient presentation lies above the abundance of those Ags, i.e. a* must be greater than *a*^*max*^. In such a case, when self-Ags and self-reactive lymphocytes are present, the APC-lymphocyte interaction is not strong enough to trigger an adaptive immune response. Note that when peripheral tolerance comes into play, the riskiest self-Ags shift from those with effective abundances around *a*^*max*^ to those around the presentation threshold a*, for which the probability of being erroneously targeted is lower.

### Correct targeting of pathogens and the risk of bystander autoimmunity

In the context of an infection, a primary goal for the AIS is correctly targeting those Ags that correspond to the pathogen, with the complication of this having to be accomplished in the presence of non-target self-Ag towards which a fraction of the immune repertoire is potentially reactive. Wrong targeting may lead to activation of self-reactive lymphocytes and eventually trigger an autoimmune response, or at least immune responses that consume resources and decrease fitness. In order to evaluate how feasible it is for the IS to achieve this goal, we introduce the correct targeting ratio, *R*, as the quotient between the probability of targeting an Ag from the pathogen and the probability of selecting one of those self-Ag for which the self-targeting risk is greatest (i.e., those self-Ag with effective abundance equal to a*). Provided that the IIS has been activated by the pathogen and the effective abundance of the target Ag is greater than the efficient presentation threshold, the correct targeting ratio *R* becomes (see Methods):

2 $$ R=\frac{p{e}^{a*{t}_0}}{\left(1-p\right)a*} $$

whereby the ratio *R* quantifies how much more probable it is for the AIS to target a pathogen Ag rather than a self-Ag. By examining its expression, it becomes evident that the greater the pathogen abundance *p*, the more easily the AIS can target it. Conversely, low concentrations of pathogens (in the context of an activated IS) are more prone to induce autoimmunity. In the least favorable scenario in terms of Ag targeting, expression (2) can be approximated by *R = e*^K s n*^, where n* is the number of MHC-peptide complexes required to activate the lymphocyte, *K* is the number of APCs that a lymphocyte scans during maturation, and *s* is the fraction of the APC surface that is scanned by the lymphocyte (see Methods). Available experimental data assign n* a typical value between 5 and 10 [28], while estimates of *K* range from a few hundred to several thousand [29]. Under the conservative assumption that lymphocytes only scan as little as a 5% of the APC surface, those parameter values render a correct targeting ratio *R* > 10^20^, which implies that Ag targeting is extremely reliable. Note that estimates of *R* become even greater if larger surface scanning fractions are considered.

In the light of our previous calculations, the wrong targeting of self-Ags that would lead to bystander activation is very unlikely, although there are two instances where that might occur with higher probability. First, wrong targeting can occur if the IS faces pathogens capable of activating the IIS while remaining at abundances too low to be detected by the AIS (i.e., below the efficient presentation threshold a*). Such a scenario trivially corresponds to a null value of the correct targeting ratio, *R* = 0, which means that the Ag targeted by the AIS, if any, will be a self-Ag. The second possibility is that the infection produces changes in the effective abundance of self-Ags, such that Ags that are usually kept out of sight of the AIS suddenly become exposed. The magnitude of this effect depends on the relative changes experienced by self-Ags. For example, if the basal abundance of a self-Ag lies around *a*^*max*^ (undetectable) and it raises to a* (detectable), the probability that it be accidentally targeted by the AIS can be as high as one third of the probability of actually targeting the pathogen. This is equivalent to a correct targeting ratio equal to three, to be compared to 10^20^ if the basal abundance of the self-Ag is already a* (detectable) but it remains unchanged during the infection. This observation suggests that autoimmunity would be more prone to occur against self-Ags the presence of which increases during certain pathologies. Indeed, this process is very similar to the mechanism through which the IS can recognize tumors, as will be explained below.

### Tumor detection

The probabilistic induction of central tolerance implies that some reactivity remains against rare self-Ags. Detecting self-Ags implies an intrinsic risk of autoimmunity but at the same time, it provides the immune system with a valuable asset that if well managed, enables it to monitor its own microenvironment and opens the door to antitumor responses. The fact that the IS can recognize tumor cells by sensing specific tumor Ags is well documented and underlies the concepts of cancer immunosurveillance and immunoediting [1,30]. We hypothesize that immune responses against tumors are based on the targeting of rare, non-tolerated self-Ags whose abundance increases during tumor progression [31,32].

Within the framework of the probabilistic IS model, there are three requisites that must be fulfilled for the IS to detect changes in the abundance of self-Ags: (1) The IS must be able to organize a response against self-Ags; (2) conserved self-reactivity must depend on the abundance of each Ag; and (3) Ag presentation must resemble Ag abundance. The first condition is an obvious pre-requisite, while the second and third conditions are necessary for meaningful Ag detection. This is a tantamount to distinguishing between noisy serendipitous activation (the risk of bystander autoimmunity) and meaningful changes in concentration. As an additional requirement, the IIS must be activated before a response can be mounted.

**Figure 3:**
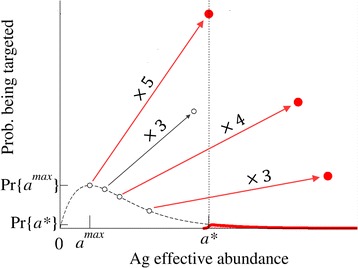
**Probability of targeting self-Ag in basal conditions (curve) and after pathological over-expression (isolated points).** The arrows indicate multiplicative changes in the effective abundances of several self-Ag (increase factors are written on the arrows). Only those Ags with effective abundances greater than the threshold *a* ^*^ can induce a response (red symbols). Note that, due to peripheral tolerance, the targeting probability for Ag with abundances below *a* ^*^ is effectively zero; the values represented in black (dashed line and open symbols) correspond to the targeting probabilities in the absence of peripheral tolerance as given by eqn. (8b). The MDI in this example has been set to 5, which means that an Ag with basal abundance equal to *a* ^*max*^ has to be over-expressed by a factor 5 in order to reach the threshold abundance *a* ^*^.

The fact that there is a threshold for efficient presentation implies that self-Ags with an effective abundance below the threshold must increase in abundance in order to induce immune responses. For example, the probability of detecting self-Ags when some of them have increased in abundance several fold can be seen in Figure 3. In basal conditions only those Ags with an abundance greater than a* (red line) can induce an auto-reactive response (natural autoimmunity), of which there are only a few and their probability of being detected is very low. In pathological conditions, changes in abundance make presentation more likely for some Ags the basal abundance of which is otherwise below a* (red points). In this way the IS can detect changes in self-Ags.

The ability of the IS to detect self-Ags that increase their abundance can be characterized through two parameters: the minimum detectable increment (MDI) and the discrimination ability (∆). We introduced the MDI to address the question of how sensitive the IS is towards changes in self-Ags. Specifically, the MDI quantifies the magnitude of the changes that the IS can detect. According to our model, the IS is not equally sensitive to changes in all self-Ags but rather, to those Ags whose basal effective abundances lie around *a*^*max*^. Thus, the MDI is defined as the factor by which the abundance of such Ags must increase in order to reach the detection threshold a*, whereby MDI = a*/a^*max*^ (see Figure 3). By using the previous result *a*^*max*^ *= 1/t*_*0*_ it is possible to express the MDI in the alternative way *MDI = t*_*0*_a*.

The discrimination ability assesses the extent to which self-recognition can be correctly interpreted as a result of a change in the self. Provided that the IIS has been previously activated by other means, detection of self-Ags by random presentation is indistinguishable from that derived from genuine changes in effective abundance. In relation to self-recognition, it is possible to solve this conundrum if it can be assumed to be more probable that a true change in concentration, rather than accidental noise-driven detection, has occurred. We define the discrimination ability (∆) as the certainty with which detection of a self-Ag can be attributed to a change in its effective abundance. The discrimination ability adopts a value between zero (when there is no certainty that detection is due to a change in abundance) and one (when the change in abundance is certain). In order to calculate it, we use the same Ag for which the MDI was defined, such that:

3 $$ \Delta =\frac{ \Pr \left\{{a}^{\max}\left(\times \mathrm{M}\mathrm{D}\mathrm{I}\right)\right\}- \Pr \left\{{a}^{\ast}\right\}}{ \Pr \left\{{a}^{\max}\left(\times \mathrm{M}\mathrm{D}\mathrm{I}\right)\right\}+ \Pr \left\{{a}^{\ast}\right\}} $$

As shown in Methods, it is possible to derive a relation that links both the MDI and the discrimination ability:

4 $$ \Delta =\frac{e^{MDI-1}-1}{e^{MDI-1}+1} $$

**Figure 4:**
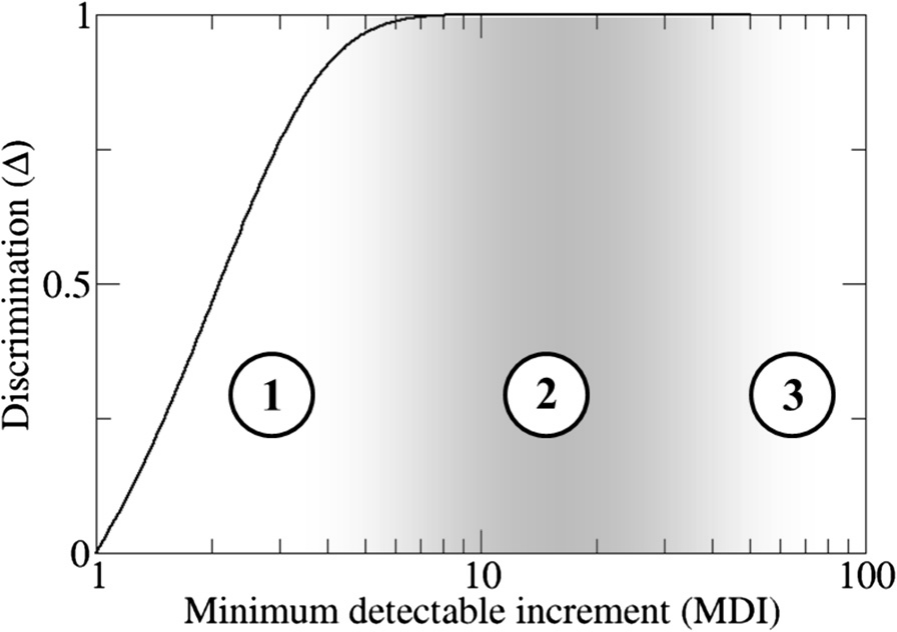
**Discrimination ability ∆ as a function of the minimum detectable increment MDI (eqn.** **3** **).** The ability of the IS to detect tumors depends on the region where it works. In region 1, the IS responds to small changes in effective abundances with low discrimination, which implies a higher risk of autoimmunity. In region 2 (shaded area), the IS responds to moderate variations in self-Ags with high discrimination making it optimal to detect tumors correctly. In region 3, large increases in effective abundances are required to make self-Ag detectable. Actually, the IS of vertebrates seems to be working in this third region. Notice that the border between regions 2 and 3 is somehow arbitrary, since a better characterization of fluctuations in Ag effective abundances would be required in order to decide what a “large” change is (see Discussion).

The dependence between ∆ and the MDI means that the IS discriminates strongly if it only responds to big changes in the self. When trying to detect small changes, the IS has to deal with a weaker capacity to distinguish between changes and random presentations. This trade-off between the MDI and discrimination can be represented (Figure 4) and the most remarkable feature is that the IS can achieve almost full discrimination by establishing its MDI just over a factor 10 (notice that this holds regardless of all other parameters of the model). An order of magnitude estimation of the actual MDI in mammals can be carried out by recalling that a*t_0_ = Ksn* (see Methods) and using for *K*, *s* and n* the same conservative estimates as in the previous section. This renders an MDI of the order of 100 (in a range from 50 to 500), which corresponds to a ∆ virtually equal to one (the difference is of the order of 10^−20^ for MDI =50 and even smaller for larger MDI). Thus, it seems that the IS of (at least) mammals works in a safety zone, where discrimination is almost perfect and detection of self-Ags requires that their effective abundances increase in a large factor.

## Discussion

The strength of an adaptive immune system lies in its ability to recognize a virtually infinite number of antigens in a highly specific manner. However, such specific recognition becomes useless, or even dangerous, if the targeted Ag is not related to the cause of IS activation. Consequently, targeting the correct Ag in a universe of self and non-self Ags, damaging and non-damaging Ags, is a fundamental task for the IS. In order to gain an intuitive understanding as to how an active IS accomplishes this task, we have explored a simple model that focuses on how the innate and adaptive components of the IS communicate with each other. The main feature of this model is that communication occurs in a probabilistic way, based on the fact that the IIS does not exactly tell the AIS which the target Ag is but rather, it presents it along with other, non-target Ags. In order to provide the AIS with the ability to discriminate Ags, our model only considers two control mechanisms: first, central tolerance, where induction is modeled as probabilistic self-Ag presentation leading to inactivation; and second, the need of a minimum number of MHC-peptide-TCR complexes to be formed in order to induce lymphocyte activation (the effective presentation threshold). Provided that the IIS only becomes activated when signals of danger are present, these simple control mechanisms are sufficient to ensure reliable targeting of the pathogen Ag by the AIS. Such a finding is striking and involves evolutionary implications that are not trivial, particularly as it shows that a very simplified IS can still be functional. It is conceivable that the addition of further complexity during evolution (e.g. regulatory feedback loops, memory, specific peripheral tolerance) will have optimized the dynamics of immune responses and enlarged their functional spectrum.

Conceptual models of the IS usually assume that every Ag present will be detected by the AIS. In the probabilistic model, we explicitly consider the possibility that rare Ags remain undetected and that if any of such Ag experiences a sudden increase in abundance it will then become visible to the IS. In the presence of activation –danger– signals, such events may lead to autoimmune or antitumor responses, the latter if a tumor Ag is involved [31,33]. Experimental evidence supporting this prediction includes detection of autoantibodies and self-reactive T-cells specific to certain types of cancer, which has been related to the overexpression of their cognate tumor-associated Ags [30,32,34,35]. It should be noted that in this model, detecting changes in Ag abundance is not based on any kind of active tracking but rather on probabilistic issues. Thus, autoimmunity and responses to tumors are two sides of the same coin: revolving around the probabilistic nature of the IIS-AIS communication. This intrinsic relation that links autoimmunity and antitumor immunity manifests itself, for instance, in similarities between the Ag involved in both kinds of responses [36,37]. In the context of immunosenescence, the model predicts that depletion of the self-reactive immune repertoire with aging would impair the IS from detecting tumors.

An order of magnitude estimation of the sensitivity of the IS to changes in self-Ag suggests that a tumor Ag’s effective abundance should increase approximately in a factor 100 in order to become detectable. While such a large change may not always be experienced by serum markers typically used for monitoring cancer [38,39], instances of cell markers whose expression in tumors raises several orders of magnitude have been reported [40]. From a systems optimization perspective, the 100-fold increase in effective abundance required for detection seems surprisingly conservative, since almost certain discrimination is compatible with detecting changes of as a small magnitude as 10-fold. A possible explanation is that ordinary fluctuations in Ag effective abundances often raise over a factor of 10, e.g. due to local changes in concentration or episodes during which they become more accessible to APCs. Therefore, the high MDI observed would contribute to filtering significant changes. Testing this idea would require a better characterization of the MHC-bound peptide profiles and their fluctuations, an information that is just starting to become available [41,42].

According to our results, the very existence of self-reactive lymphocytes can be at least partially explained by the probabilistic nature of Ag presentation during lymphocyte maturation. Complete tolerance cannot be achieved since the period of maturation is limited. This constraint on central tolerance would still hold even if all self-Ags were expressed in the thymus and therefore, incomplete tolerance and residual self-reactivity are not faults but intrinsic properties of the vertebrate IS. In our model the mechanism of peripheral tolerance is very basic, static and unspecific, and could be considered a sort of ‘peripheral ignorance’. However, it is easy to envisage what would happen with a more sophisticated mechanism able to induce tolerance to those Ags that are efficiently presented in the absence of activation signals. In such circumstances, increases in the abundance of rare Ags would switch them from untolerated to tolerated, and such specific peripheral tolerance would have dramatic consequences in terms of tumor detection. Indeed, the lack of activating signals may allow tumors to escape the IS and induce tolerance to the over-expressed tumor Ags [43]. This represents an instance of how a mechanism evolved to reduce the risk of autoimmunity entails a cost at the level of tumor detection.

A connection between genes involved in Ag presentation and susceptibility to autoimmune diseases has already been suggested on the basis of the results of genome wide association studies [44,45]. The probabilistic model of the IS in turn captures some of the mechanisms through which infections can trigger autoimmune responses [21], identifying two risk factors of interest. First, the risk of an autoimmune response increases in the presence of pathogens that activate the IIS while remaining at concentrations too low to be effectively presented to the AIS. The second risk factor is the massive release of otherwise rare self-Ags as a result of infection. In both cases, autoimmunity would follow from incorrect Ag targeting and its onset might possibly require additional failings of other regulatory mechanisms not included in our model [46]. An experimental setting that simulates the aforementioned scenario is the induction of experimental autoimmune encephalomyelitis, an animal model of multiple sclerosis, after priming with central nervous system-restricted Ags plus an adjuvant [47]. Alternatively, autoimmunity can follow massive Ag release after traumatic lesions provided that they lead to immune activation. This may be the case of sympathetic ophthalmia, a rare autoimmune pathology that affects both eyes after accidental or surgical trauma in one of the eyes [48].

From the point of view of comparative immunology, the results we have obtained with this simple model provide hints as to what might be expected when studying different immune systems. Incomplete tolerance, some risk of autoimmunity and the ability to detect tumors are intrinsic properties of immune systems whose innate and adaptive components communicate probabilistically. Nevertheless, it is worth noting that there are alternatives to probabilistic communication. For instance, the intracellular RNA interference system of plants detects and processes viral double-stranded RNA to produce target-specific effector molecules (siRNA) [49]. From a conceptual perspective, the RNA interference system involves innate –dsRNA recognition– and adaptive –effector siRNA– components that communicate deterministically through RNA processing. Alternatively, immune systems with no clear distinction between innate and adaptive components may also behave as a probabilistic IS if: (1) their highly diversified receptors are potentially capable of binding self-molecules; and (2) they respond to activation (danger) signals that are not physically linked to their “target Ags”. As research on the immunology of jawless vertebrates, protochordates and invertebrates progresses [2,8,50,51], it will be of great interest to see whether their highly diversified immune systems are based on, and share, the characteristics of those with probabilistic communication strategies [18]. If that is the case, we expect that the general conclusions obtained in this work will also hold for them.

## Conclusion

A simple probabilistic model of the communication between the innate and adaptive immune system provides a robust immune response, including targeting tumors, but at the price of being at risk of developing autoimmunity.

## Methods

### Effective abundance

As explained above, our model deals with an IS immersed in a complex antigenic universe. Each Ag *A*_*i*_ is characterized by its effective abundance *a*_*i*_, which is a normalized quantity accounting for the probability that *A*_*i*_ is presented by APCs. Ideally, the effective abundance would be the product of the actual abundance, the degree of access that APCs have to the Ag, and the affinity of the MHC-peptide complex, normalized in such a way that the sum of effective abundances for all Ags equals one. According to that, the effective abundance increases not only if the actual abundance does, but also if the Ag becomes more accessible to the APCs (e.g. after infiltration of APCs in a tissue), or if a particular MHC allele has greater affinity for that Ag.

### Self-reactivity of the immune repertoire after thymic negative selection

Central tolerance was modeled as a stochastic process that mimics thymic negative selection on the immune repertoire. To that end, we first calculated the probability that a self-reactive lymphocyte finds its cognate Ag during maturation and experiences negative selection. The maturation process itself is modeled as a number *t*_*0*_ of Ag presentations, where the probability of antigen *A*_*i*_ being presented is proportional to its effective abundance *a*_*i*_. For this process, the probability that *A*_*i*_ is presented *k* times to the self-reactive lymphocyte follows a binomial distribution:

5 $$ \Pr \left\{{A}_i\;\mathrm{presented}\;k\;\mathrm{times}\right\}=\left(\begin{array}{c}\hfill {t}_o\hfill \\ {}\hfill k\hfill \end{array}\right){a}_i^k{\left(1-{a}_i\right)}^{t_o-k} $$

Provided that the number of Ag presentations during lymphocyte maturation is large, the binomial distribution can be approximated by a Poisson distribution. Therefore, the previous probability becomes:

6 $$ \Pr \left\{{A}_i\;\mathrm{presented}\;k\;\mathrm{times}\right\}\approx \frac{{\left({a}_i{t}_o\right)}^k}{k!}{e}^{-{a}_i{t}_o} $$

The probability that a self-reactive lymphocyte escapes negative selection can be obtained by making *k* =0. If there are *m* naive lymphocytes reactive to the same self-Ag, all of them must experience negative selection so that the self-Ag becomes tolerated. That would happen with probability *f*_*i*_, whereby:

7 $$ {f}_i= \Pr \left\{{A}_i\;\mathrm{tolerated}\right\}={\left(1-{e}^{-{a}_i{t}_o}\right)}^m $$

which, for *m* =1, is the expression reported in the Results section as eqn. (1).

In the context of an ongoing infection, there is a possibility that self-antigens which are not tolerated be erroneously targeted by the IS. For a given self-Ag, the probability of being targeted is proportional to its effective abundance and conditional to not being tolerated. In mathematical terms, that can be expressed as the product of the effective abundance and the probability of not being tolerated. If there are *m* self-reactive clones per each self-Ag the targeting probability becomes:

8a $$ \Pr \left\{{A}_i\ \mathrm{targeted}\right\} = 1-{\left(1-{a}_i\ \left(1-{f}_i\right)\right)}^m $$

In the simplest case with *m* =1, equation (8a) takes a more compact form:

8b $$ \Pr \left\{{A}_i\ \mathrm{targeted}\right\} = {a}_i{e}^{-{a}_i{t}_0} $$

The probability of being targeted is maximum for self-Ag with effective abundance equal to *a*^*max*^. The exact expression for *a*^*max*^ can be obtained by taking the derivative of equation (8) with respect to *a*_*i*_ and making it equal to zero. This results in *a*^*max*^ = 1/*t*_0_*m*.

### Effective abundance threshold for efficient Ag presentation

In order to incorporate a simple form of peripheral tolerance in our model, we introduce the following premise: lymphocyte activation by APCs requires the formation of a minimum number n* of MHC-peptide-TCR complexes in the surface of the APC. In other words, only those antigens that appear in (at least) n* MHC-peptide complexes on the same APC will be able to induce a response. For an antigen *A*_*i*_ with effective abundance *a*_*i*_, the probability that this happens is equal to:

9 $$ \Pr \left\{{A}_i\ \mathrm{represented}\ \mathrm{at}\ \mathrm{least}\ {n}^{*}\ \mathrm{times}\right\}=1-{e}^{-{a}_iN}{\displaystyle \sum_{j=0}^{n^{*}-1}}\frac{\left({a}_i{N}^j\right)}{j!} $$

where *N* is the total number of MHC-peptide complexes on the surface of the APC. Provided that *N* is moderately large, this expression grows rapidly from zero to one when the effective abundance reaches a value a* = n*/*N*. An example of this behavior is shown in Figure 2 (middle panel). The effective abundance a* can be interpreted as a threshold for efficient Ag presentation.

Once peripheral tolerance is taken into account, the probability of targeting a given self-Ag can be calculated by multiplying the expressions in equations (8a) and (9). This is how the solid curve in the lower panel of Figure 2 was obtained. However, for practical purposes equation (9) can be approximated by a Heaviside step function centered in a*. Under that approximation, the probability of targeting a self-Ag becomes:

10 $$ \Pr\ \left\{{A}_i\ \mathrm{targeted}\right\} = \left\{\begin{array}{cc}\hfill 0\hfill & \hfill \mathrm{if}\ {a}_i<{a}^{*}\hfill \\ {}\hfill 1-{\left(1-{a}_i\ \left(1-{f}_i\right)\right)}^m\hfill & \hfill \mathrm{if}\ {a}_i\ge {a}^{*}\hfill \end{array}\right. $$

Notice that, provided that a* > *a*^*max*^, the probability of being targeted is maximum for those self-Ag with effective abundance equal to a*.

### Pathogen vs. self-Ag targeting

We introduce the correct targeting ratio, *R*, as a tool to evaluate the performance of the IS when it comes to targeting pathogen Ag. *R* is defined as the quotient between the probabilities of targeting an antigen from the pathogen and a self-Ag with effective abundance a* (notice that those are the self-Ags for which the self-targeting probability is greatest). In order to compute *R* it must be recalled that the presence of a pathogen modifies the effective abundances of self-Ag, multiplying them by a factor (1 – *p*), were *p* is the effective abundance of pathogen Ag (this comes from the fact that effective abundances are normalized). As we work with normalized effective abundances, the probability of targeting an antigen from the pathogen is simply equal to the effective abundance *p*. Moreover, we assume that *p* ≥ a*, so that the pathogen is efficiently presented (otherwise, the AIS would be unable to “see” the pathogen). Taking all that into account, the correct targeting ratio becomes:

11 $$ R=\frac{ \Pr \left\{\mathrm{Pathogen}\ \mathrm{targeted}\right\}}{ \Pr \left\{{A}^{*}\ \mathrm{targeted}\right\}}=\frac{p}{\left(1-p\right)\left[1-{\left(1-{a}^{*}\left(1-{f}^{*}\right)\right)}^m\right]}\approx \frac{p\ {e}^{a^{*}{t}_0}}{\left(1-p\right)\ {a}^{*}\ {m}^2} $$

The last approximation has been obtained by discarding terms of lower order in $\left({e}^{a^{*}{t}_0}\right)$. Biological data allow to estimate the order of magnitude of this factor around 10^20^ (see Results) which supports the validity of the truncation. In the particular case *m* =1 equation (2) reported in the Results section is exactly recovered.

A lower bound for *R* can be calculated if the concentration of pathogen is as low as the minimum detectable, *p* = a*. In this least favorable scenario, and provided that most Ag effective abundances (a* among them) are much smaller than one, the correct targeting ratio can be approximated by $R\approx {e}^{a_i{t}_0}$. Let us denote by *K* the number of APCs that a lymphocyte scans during maturation and *s* the fraction of the APC surface scanned by the lymphocyte. Then it is possible to express the number of presentations during maturation as *t*_0_ = *KNs*, which combined with a* = n*/*N* renders $R\approx {e}^{Ks{n}^{*}}$.

### Detection of tumors by the IS

Because the probability of targeting an Ag depends on its effective abundance, changes in Ag abundances modify targeting probabilities. Specifically, let us suppose that the effective abundance of antigen *A*_*i*_ experiences a *k*-fold increase. Then the targeting probability for that Ag increases from the value in equation (10) to the new value:

12 $$ \Pr\ \left\{{A}_i\ \left(\times k\right)\ \mathrm{targeted}\right\} = \left\{\begin{array}{cc}\hfill 0\hfill & \hfill \mathrm{if}\ k{a}_i<{a}^{*}\hfill \\ {}\hfill 1-{\left(1-k{a}_i\ \left(1-{f}_i\right)\right)}^m\hfill & \hfill \mathrm{if}\ k{a}_i\ge {a}^{*}\hfill \end{array}\right. $$

Two observations can be made from this expression. First, the IS is not equally sensitive to changes in all self-Ag: by taking the derivative with respect to *k* and finding its maximum it comes out that the IS is most sensitive to changes in self-Ag whose basal effective abundances are equal to *a*^*max*^ = 1/*t*_0_ (notice that, for *m* =1, that coincides with the Ag for which the targeting probability in the absence of peripheral tolerance is greatest). The second observation is that such Ag will be only detected if its effective abundance becomes greater than a*, i.e. if *k* ≥ a*/*a*^*max*^. The value of *k* such that *ka*^*max*^ = a* defines a characteristic factor that we termed Minimum Detectable Increment (MDI). By using previous results, it is possible to express it as MDI = a*t_0_.

The discrimination ability (∆) is introduced with the aim of evaluating the performance of the IS when trying to detect tumors. It quantifies the certainty with which detection of a self-Ag can be attributed to a real increase in its effective abundance and not just to chance. In order to calculate ∆ we compare the probability of targeting an Ag with basal abundance *a*^*max*^ that has experienced an increase of the magnitude of the MDI with the probability of targeting an Ag with basal abundance a^*^ (the most likely to be targeted in the absence of changes). Let us denote those probabilities Pr{*a*^*max*^(×MDI)} and Pr{a*}, respectively.

$$ \begin{array}{l} \Pr \left\{{a}^{max}\left(\times \mathrm{M}\mathrm{D}\mathrm{I}\right)\right\}=1-{\left[1-{a}^{*}\left(1-{\left(1-{e}^{-1}\right)}^m\right)\right]}^m\hfill \\ {} \Pr \left\{{a}^{*}\right\}=1-{\left[1-{a}^{*}\left(1-{\left(1-{e}^{-{a}^{*}{t}_0}\right)}^m\right)\right]}^m\hfill \end{array} $$

The discrimination ability is defined as:

and after some manipulations it becomes

13 $$ \Delta =\frac{\delta -1}{\delta +1}. $$

Where

$$ \delta \approx \frac{1-{\left(1-{e}^{-1}\right)}^m}{1-{\left(1-{e}^{-\mathrm{M}\mathrm{D}\mathrm{I}}\right)}^m} $$

The approximation has been done by discarding terms of order (a*)^2^ and higher. In the particular case *m* = 1 this expression is exact and can be expressed as

Notice that, because of its definition, ∆ takes values between zero (detecting “noise” is as probable as detecting changes) and one (detection is always due to meaningful changes). The relation between ∆ and the MDI does not depend on any of the parameters of the model (a_*_, *t*_*0*_).

## Abbreviations

Ag: Antigen
AIS: Adaptive immune system
APC: Antigen presenting cell
dsRNA: double-stranded RNA
IIS: Innate immune system
IS: Immune system
MDI: Minimum detectable increment
MHC: Major histocompatibility complex
siRNA: Small interference RNA
TCR: T cell receptor
